# Multifocal Pigmented Villonodular Synovitis in the Noonan Syndrome

**DOI:** 10.1155/2018/7698052

**Published:** 2018-12-06

**Authors:** Othmane Miri, Nicolas Bonnet, Philippe Lysy, Naima Loucheur, René Gayito, Pierre Louis Docquier

**Affiliations:** ^1^Cliniques Universitaires Saint-Luc, Service d'Orthopédie et de Traumatologie de l'Appareil Locomoteur, Avenue Hippocrate 10, B-1200 Brussels, Belgium; ^2^Cliniques Universitaires Saint-Luc, Service d'Endocrinologie Pédiatrique, Avenue Hippocrate 10, B-1200 Brussels, Belgium; ^3^Université Catholique de Louvain, Secteur des Sciences de la Santé, Institut de Recherche Expérimentale et Clinique, Neuro Musculo Skeletal Lab (NMSK), Avenue Mounier 53, B-1200 Brussels, Belgium

## Abstract

Noonan-like/multiple giant cell lesion (NS/MGCL) is a rare condition overlapping with Noonan syndrome. Once thought to be a specific and separate entity, it is now suggested to be a variant of the Noonan syndrome spectrum. We report the case of an 8-year-old boy with a typical clinical picture of Noonan syndrome with a de novo germline mutation of PTPN11 (c.854 T>C). During his follow-up, the patient developed multifocal pigmented villonodular synovitis which first affected the left knee and shortly after both elbows.

## 1. Introduction

Noonan syndrome (NS) is a genetic disorder, with an autosomal dominant inheritance pattern. The cardinal features are craniofacial dysmorphism, short neck with webbing, skeletal malformations, heart defects such as pulmonary stenosis and hypertrophic cardiomyopathy, short stature, cryptorchidism, and bleeding disorders [1]. The incidence reported is approximately 1 in 2500 people, but it is likely to be higher due to the high variability of the phenotype [2]. Noonan syndrome is subjected to genetic heterogeneity. Mutations in multiple genes (PTPN11, SOS1, RAF1, RIT1,…) can actually cause this condition [3]. These genes provide instructions for making proteins that are important in the RAS/MAPK cell signalling pathway, which is needed for the regulation of cell growth and division. Mutations in PTPN11 gene are responsive for about half of the cases [3].

Pigmented villonodular synovitis (also called tenosynovial giant cell tumor) is a rare benign proliferative articular pathology poorly understood, characterized by an abnormal proliferation of the synovial membrane. It can affect all joints but most frequently the large ones (knee, hip, ankles …). These lesions consist of multinucleated giant cells in a background of fibrous connective tissue with mononucleated cells, among which were hemosiderin-laden macrophages. The clinical picture includes chronic swelling and joint pain. Ultrasound is generally the first imaging exam, but MRI represents the best imaging modality to explore this disease. Histopathology of the synovial tissue is always mandatory to make the definitive diagnosis. In case of delayed diagnosis, this condition could lead to joint destruction. The management of PVS consists of radical synovectomy but is often difficult because of the high risk of relapse.

In 1991, Cohen and Gorlin [4] were the first to describe a patient with the association of the typical NS phenotype and the MGCL and named it Noonan-like/multiple giant cell lesion syndrome. There is actually no recorded incidence due to the scarcity of these diseases and thus their association.

## 2. Case Presentation

An 8-year-old boy with Noonan syndrome, genetically confirmed by a de novo germline heterozygous mutation of PTPN11 (c.854 T>C), was referred for a swelling of the left knee.

His medical history was marked by a neonatal hypertrophic cardiomyopathy and pulmonary valve stenosis (which needed balloon dilatation procedures and surgery), a transcatheter closure of an atrial septal defect, an orchidopexy for right cryptorchidism, a right renal malrotation, a percutaneous endoscopic gastrostomy for eating disorders during 4 years, a slight delay of language, a cystic lymphatic malformation of the right chest wall, and finally, a short stature which required a growth hormone treatment for a sustainable growth.

**Figure 1:**
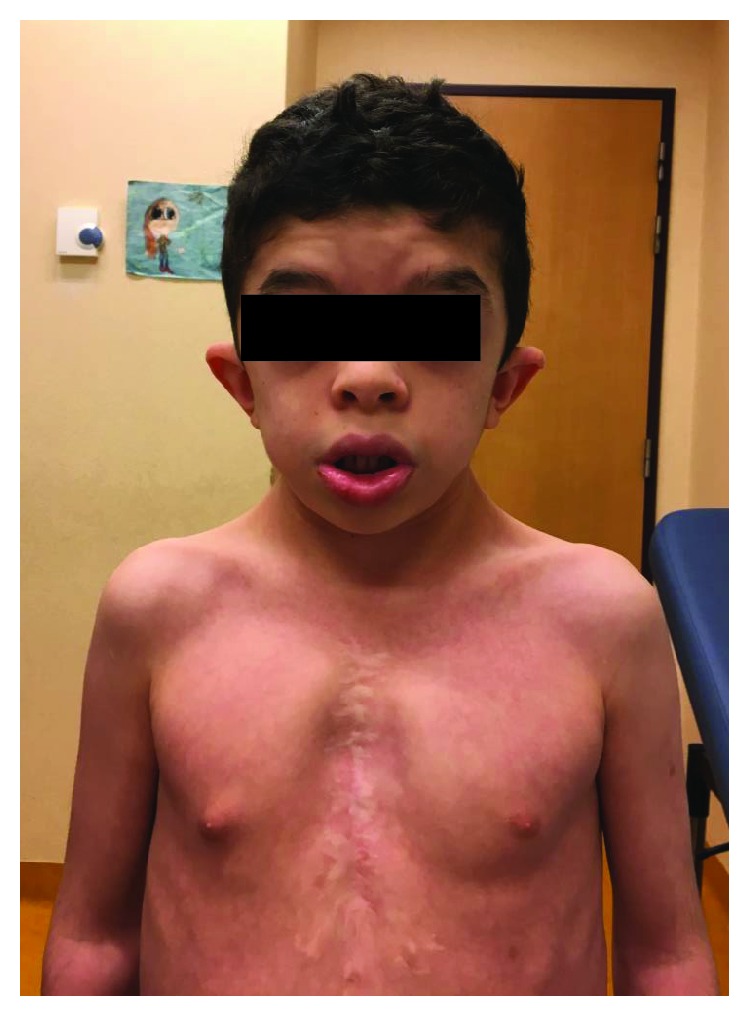
An 8-year-old patient with small hypertelorism, mild ptosis, downslanting palpebral fissures, low-set and posteriorly angulated ears, high arched palate, short neck, and pectus excavatum.

The clinical examination revealed classical features of this syndrome including small hypertelorism, mild ptosis, downslanting palpebral fissures, low-set and posteriorly angulated ears, high arched palate, short neck, and pectus excavatum (Figure 1). We also noticed an extensive swelling on the lateral side of his left knee without any functional disability or pain.

**Figure 2:**
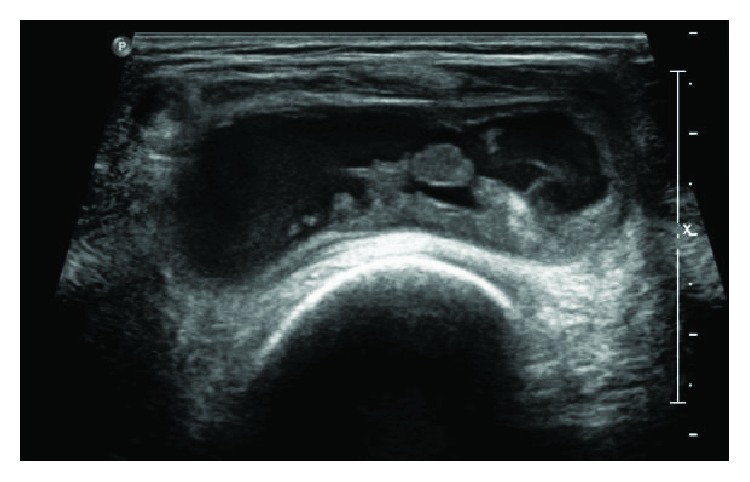
Ultrasounds of the left knee joint showing a joint effusion with villous hyperplasia of the synovial lining.

**Figure 3:**
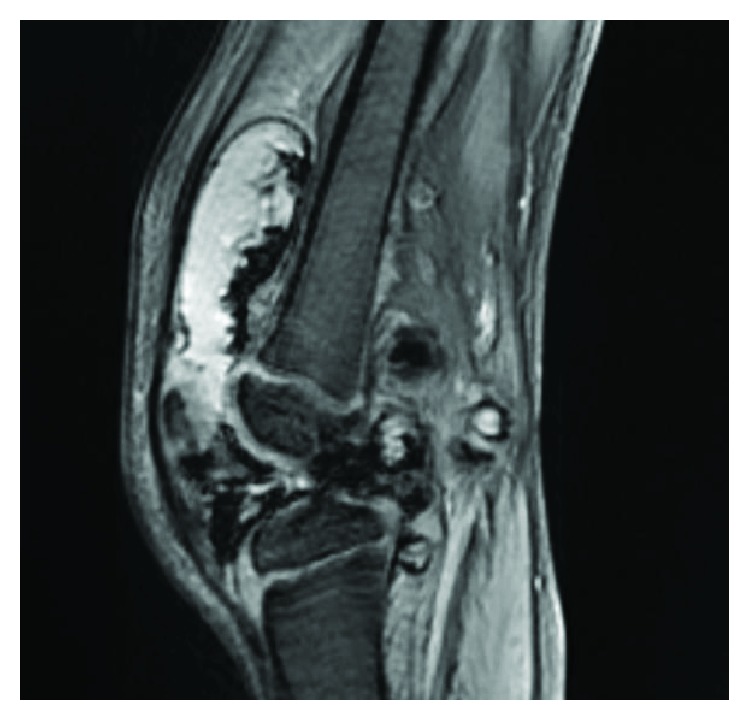
MRI, T2 sequence of the left knee: important joint effusion.

**Figure 4:**
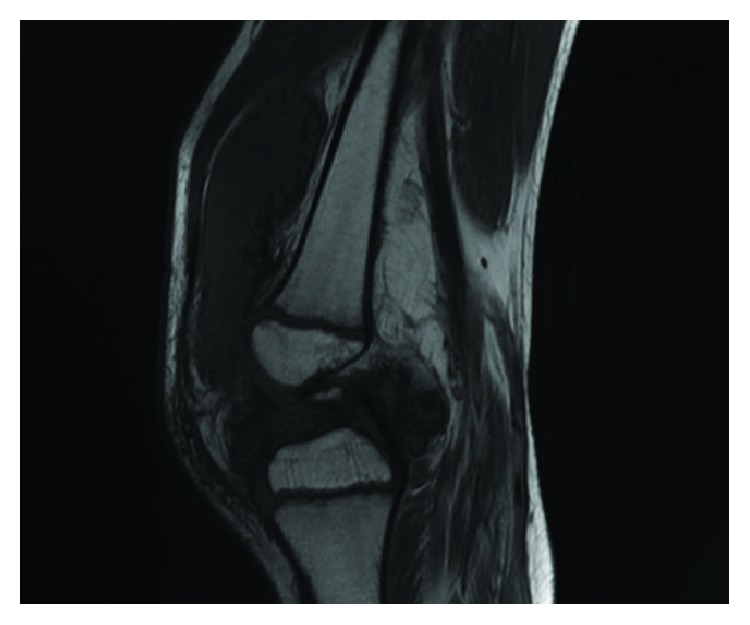
MRI, T1 sequence of the left knee: visible villonodular thickening of the synovial membrane with hemosiderin deposition.

An ultrasound imaging showed a joint effusion with villous hyperplasia of the synovial lining in his posterior part (Figure 2). The patient underwent a joint puncture, which revealed hemarthrosis. Diagnosis of PVS was then suspected by magnetic resonance imaging based on an important joint effusion (Figure 3) and villonodular thickening of the synovial membrane with hemosiderin deposition (Figure 4).

Arthroscopic total synovectomy was successfully performed. Histopathology of the synovial tissue showed villous hyperplasia on macroscopic examination and multinucleated giant cells in a background of fibrous connective tissue with numerous blood vessels and inflammatory infiltrates with hemosiderin-laden macrophages on microscopic examination. The patient recovered a complete mobility of his knee with intensive physiotherapy.

**Figure 5:**
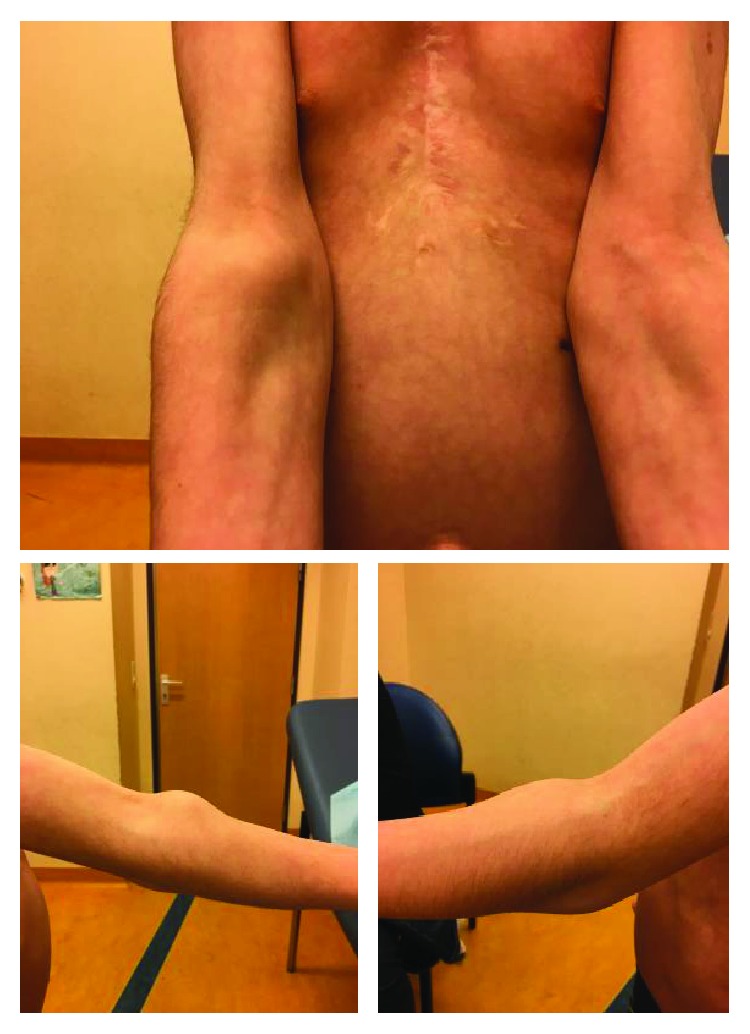
Clinical imaging showing the swelling around the elbow joint.

Almost one year later, he developed a painless tumefaction of the anterior surface of both elbows (Figure 5). Considering his medical history, the diagnosis of PVS was mentioned again and was suspected by the MRI. A bilateral total surgical synovectomy was performed in a single-stage procedure, as the main part of the PVS was extra-articular, thereby limiting the use of arthroscopy. The patient had a complete recovery of his elbows' functional abilities.

At this time, no relapses have occurred on his left knee or both elbows and we did not notice any new lesion.

## 3. Discussion

Pigmented villonodular synovitis (PVS) was first described by Jaffe et al. [5] in 1941. This benign condition, which can involve the joints or the tendon sheaths, is characterized by the proliferation of the synovial tissue associated with hemosiderin deposition inside the synovial lining. It can be locally aggressive as it can lead to joint destruction in the absence of adequate treatment due to repeated hemarthrosis [6]. The treatment consists of arthroscopic or open surgical total synovectomy of the joint with sometimes an adjuvant radiotherapy [7].

When Cohen and Gorlin [4] described for the first time the Noonan-like/multiple giant cell lesion syndrome, this pathology was considered as a specific and separate entity from the NS but recent genetic and clinical studies [8–10] seem to indicate that it is a variant of the NS spectrum.

**Figure 6:**
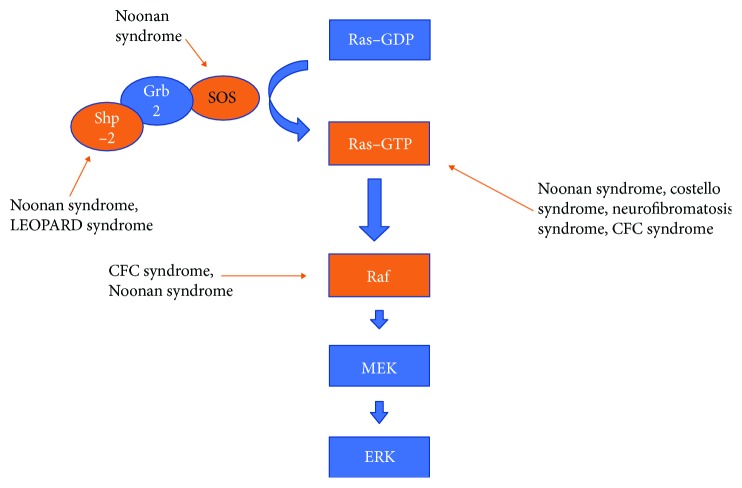
Summary of the Ras/MAPK cell signalling pathway and relation with some syndromes (nonexhaustive).

As described above, Noonan syndrome is an autosomal dominant condition, which is subjected to genetic heterogeneity. This developmental disorder is linked to mutations of genes (PTPN11, SOS1, RAF1 …) encoding various components of the RAS/MAPK cell signalling pathway. This signalling cascade is also involved in many other inherited conditions such as cardiofaciocutaneous syndrome (CFCS), LEOPARD syndrome (LS), Costello syndrome (CS), and type 1 neurofibromatosis (NF1) [11] (Figure 6). All of these syndromes have already been associated with the development of single or multiple giant cell lesions, either in the form of bone giant cell lesion (e.g., jaw involvement) or pigmented villonodular synovitis. As numerous genes have been involved, there is currently no genotype-phenotype correlation. It suggests that dysregulation of the RAS/MAPK cell signalling cascade is the basic molecular event predisposing to giant cell lesion formation rather than a specific mutation effect.

In the vast majority of the case reports of the association of NS-PVS, the same mutation in the PTPN11, SOS1, RAF1, BRAF1, and MAP2K1 genes was noticed [12].

In the literature, there are many case reports of association between NS and mandibular MGCL [12], but there are fewer reports of association between NS and PVS (only 7 cases of NS-PVS association were reported by Cohen and Gorlin [4]). Beneteau et al. [13] reported five cases of NS with MGCL or PVS and showed that not only PTPN11 but also SOS1 mutation was responsible for the syndrome. Of these five patients, only one presented PVS of the ankle. In 2008, Mascheroni et al. [14] reported one case of a 13-year-old boy with NS and PVS of the right ankle. Diagnosis was settled after several months of pain and tumefaction of the ankle (Table 1).

## 4. Conclusion

In conclusion, genetic and clinical studies have strongly suggested the link between NS and PVS. A study should be carried out to highlight the link between NS and PVS. This study would lead to a better knowledge of the pathology and faster treatment.

It is important to rule out PVS in case of joint pain or swelling in Noonan syndrome patients. As PVS is a potentially locally aggressive pathology, treatment with synovectomy is mandatory. As seen in our case report, a fast management of the PVS is needed to avoid the potential major complication as cartilage damage due to repeated hemarthrosis.

The follow-up of people with NS should be carried out by specialized teams with a thorough knowledge of these rare disorders.

## Figures and Tables

**Table 1:** Late reported pigmented villonodular synovitis.

Title	Author	Revue	Year	Noonan syndrome	Gene involved	MRI	Histology: MGCL in cellular fibrous tissue	Localisation	Treatment
“Pigmented Villonodular Synovitis of the Knee Joint: A Case Report” [7]	Kapoor et al.	Open Access Case Knee Joint: A Case Report	2016	—	NA	+	+	Left knee	Subtotal synovectomy
“SOS1 and PTPN11 Mutations in Five Cases of Noonan Syndrome with Multiple Giant Cell Lesions” [13]	Beneteau et al.	European Journal of Human Genetics	2009	+	SOS1	+	+	Left ankle	NA
“Pigmented Villonodular Synovitis in a Patient with Noonan Syndrome and SOS1 Gene Mutation” [14]	Mascheroni et al.	American Journal of Medical Genetics	2008	+	SOS 1 (R552S mutation)	+	+	Right ankle	Synovectomy

NA: information not available; MRI: magnetic resonance imaging; MRI +: nodular synovial proliferation with deposit of hemosiderin and joint effusion.
